# Distribution of 2,4-Diacetylphloroglucinol Biosynthetic Genes among the *Pseudomonas* spp. Reveals Unexpected Polyphyletism

**DOI:** 10.3389/fmicb.2017.01218

**Published:** 2017-06-30

**Authors:** Juliana Almario, Maxime Bruto, Jordan Vacheron, Claire Prigent-Combaret, Yvan Moënne-Loccoz, Daniel Muller

**Affiliations:** Centre National de la Recherche Scientifique, Institut National de la Recherche Agronomique, Université de Lyon, Université Claude Bernard Lyon1, VetAgro Sup, UMR Ecologie MicrobienneVilleurbanne, France

**Keywords:** DAPG, 2,4-diacetylphloroglucinol, *phl* Operon, biocontrol agent, *phlACBD* genes

## Abstract

Fluorescent pseudomonads protecting plant roots from phytopathogens by producing 2,4-diacetylphloroglucinol (DAPG) are considered to form a monophyletic lineage comprised of DAPG^+^ *Pseudomonas* strains in the “*P. corrugata*” and “*P. protegens*” subgroups of the “*Pseudomonas fluorescens*” group. However, DAPG production ability has not been investigated for many species of these two subgroups, and whether or not the DAPG^+^ *Pseudomonas* are truly monophyletic remained to be verified. Thus, the distribution of the DAPG biosynthetic operon (*phlACBD* genes) in the *Pseudomonas* spp. was investigated in sequenced genomes and type strains. Results showed that the DAPG^+^ *Pseudomonas* include species of the “*P. fluorescens*” group, i.e., *P. protegens, P. brassicacearum, P. kilonensis*, and *P. thivervalensis*, as expected, as well as *P. gingeri* in which it had not been documented. Surprisingly, they also include bacteria outside the “*P. fluorescens”* group, as exemplified by *Pseudomonas* sp. OT69, and even two Betaproteobacteria genera. The *phl* operon-based phylogenetic tree was substantially congruent with the one inferred from concatenated housekeeping genes *rpoB, gyrB*, and *rrs*. Contrariwise to current supposition, ancestral character reconstructions favored multiple independent acquisitions rather that one ancestral event followed by vertical inheritance. Indeed, based on synteny analyses, these acquisitions appeared to vary according to the *Pseudomonas* subgroup and even the phylogenetic groups within the subgroups. In conclusion, our study shows that the *phl*^+^ *Pseudomonas* populations form a polyphyletic group and suggests that DAPG biosynthesis might not be restricted to this genus. This is important to consider when assessing the ecological significance of *phl*^+^ bacterial populations in rhizosphere ecosystems.

## Introduction

About a century ago, the *Pseudomonas* genus was defined as a bacterial group gathering all aerobes, bacilli, and Gram-negative bacteria (Migula, 1894). The *Pseudomonas* classification has since considerably evolved through molecular phylogenetics, and phylogenies obtained using the *rrs* gene encoding 16S rRNA allowed the reclassification of many species into other bacterial genera (Brosch et al., 1996; Grimont et al., 1996; Anzai et al., 2000). Other molecular markers were also used to trace the evolutionary history of *Pseudomonas*, such as *rpoB, rpoD*, and *gyrB* genes (Ait Tayeb et al., 2005). In recent times, the use of concatenated phylogenetic markers or complete genome sequences has enabled a robust delimitation of the *Pseudomonas* genus (Yamamoto et al., 2000; Hilario et al., 2004; Frapolli et al., 2007; Mulet et al., 2010; Gomila et al., 2015; Garrido-Sanz et al., 2016), which encompasses several groups of closely-related species, such as the “*P. fluorescens*” group, “*P. putida*” group, “*P. aeruginosa*” group, “*P. stuzeri*” group, and “*P. syringae”* group.

Numerous *Pseudomonas* species or strains are presenting specific interactions with their eukaryotic hosts, such as humans (many strains belonging to the “*P. aeruginosa*” group), fungi (strains belonging to the species *Pseudomonas tolaasii*), or plants (many strains from the “*P. fluorescens*” group) (Munsch et al., 2000; Finnan et al., 2004; Mithani et al., 2010). Interaction with plants may involve (i) deleterious effects in the case of *Pseudomonas* plant pathogens, such as strains belonging to the species *P. syringae, P. corrugata*, or *P. mediterranea* (Catara et al., 2002; O'Brien et al., 2011; Trantas et al., 2015), or (ii) beneficial effects (Mirza et al., 2006) especially by protecting plants against a wide range of plant pathogens or pests (Mazurier et al., 2009; Almario et al., 2013a; Kupferschmied et al., 2013) through the production of many secondary metabolites such as 2,4-diacetylphloroglucinol (DAPG). This compound was also involved in the elicitation of plant defenses through induced systemic resistance (Iavicoli et al., 2003; Weller et al., 2012) and directly in the modulation of plant hormonal balance by acting as an auxin-mimetic compound (Brazelton et al., 2008).

DAPG synthesis has been so far associated with the presence of the *phl* gene cluster, comprising the *phlACBDE* operon and other genes such as *phlF, phlG, phlH*, and *phlI*. The DAPG synthesis probably starts with the type III polyketide synthase (encoded by gene *phlD*), which allows the cyclization of malonyl-CoA to phloroglucinol (Achkar et al., 2005). Then, a monoacetylphloroglucinol (MAPG) acetyltransferase (encoded by *phlACB*) acetylates phloroglucinol to MAPG and then DAPG (Kidarsa et al., 2011). Besides the *phlACBD* genes directly involved in DAPG biosynthesis, the last gene of the *phl* operon (*phlE*) is involved in the efflux of DAPG (Abbas et al., 2004). Next to the *phl* operon, *phlF* and *phlH* code for transcriptional regulators, involved respectively in the repression and activation of DAPG production (Schnider-Keel et al., 2000). Gene *phlG* encodes a hydrolase allowing a return of DAPG to MAPG (Bottiglieri and Keel, 2006). The role of *phlI* (encoding a hypothetical protein) in DAPG production is still unknown (Hayashi et al., 2012).

DAPG^+^ *Pseudomonas* were isolated around the world and exhibited a cosmopolitan distribution (Wang et al., 2001; Almario et al., 2013b, 2014; Vacheron et al., 2016). They are ecologically important as they can control phytopathogens and contribute to disease suppressiveness (Sanguin et al., 2008; Almario et al., 2013a). Multilocus sequence analysis based on housekeeping genes classified them into six main phylogenetic groups noted A to F (Frapolli et al., 2007, 2012), belonging to the “*P. corrugata*” subgroup (multilocus phylogenetic groups A to E) and the “*P. protegens*” subgroup (multilocus phylogenetic group F) of the “*P. fluorescens*” group defined by Mulet et al. (2010). DAPG production is thus documented in some species of the “*P. corrugata*” subgroup (like *P. brassicacearum*) and “*P. protegens*” subgroup (like *P. protegens*), but it has not been investigated extensively in many species of these subgroups. Consequently, it is unclear whether or not *phl*^+^ *Pseudomonas* form a monophyletic group within the “*P. fluorescens”* group, as it was claimed (Moynihan et al., 2009). Similarly, the ability to produce DAPG has been proposed as a highly conserved trait (Moynihan et al., 2009), but again it was based on a rather limited number of species and genomes. Recent progress in the sequencing of *Pseudomonas* genomes, the phylogenetic assessment of *phl*^+^ *Pseudomonas* (Frapolli et al., 2012) and the description of the *Pseudomonas* genus (including new species and the recognition of “*P. protegen*s” as a new subgroup; Gomila et al., 2015; Garrido-Sanz et al., 2016, 2017) make it now possible to conduct a more global assessment of this issue.

The aim of this study was to assess the distribution of the DAPG biosynthetic operon (*phlACBD*) among sequenced *Pseudomonas* genomes including some type strains. The occurrence of DAPG production ability in *Pseudomonas* spp. was determined, based on presence of the *phlACBD* operon and confirmatory HPLC analyses, and the phylogenies of *phl*^+^ and *phl*^−^ *Pseudomonas* spp. were compared. In addition, the genomic context of the *phlACBD* operon in the *phl*^+^ *Pseudomonas* genomes available was further explored.

## Materials and methods

### Cultivation of *Pseudomonas* type strains and DNA extraction

The 12 *Pseudomonas* type strains (Table S1) from the “*P. corrugata,” “P. chlororaphis,”* and “*P. fluorescens”* subgroups were routinely grown on Luria-Bertani agar (Sambrook et al., 1989). For genomic DNA extraction, bacterial strains were grown overnight with shaking (150 rpm) in 20 mL of liquid LB medium, and DNA was extracted from 500 μL of bacterial culture using the NucleoSpin Tissue kit (Macherey-Nagel, Hoerdt, France), following the manufacturer's instructions. DNA was quantified spectrophotometrically and adjusted to 30 ng μL^−1^.

### Search for *phl* genes in *Pseudomonas* type strains

The search for the *phl* operon (*phlACBD* genes) in genome-sequenced *Pseudomonas* type strains was carried out by BlastN using *phlACBD* genes sequences of *P. kilonensis* F113 as query. Blast parameters were fixed at 90% coverage and 75% identity in gene sequences (E < 10^−20^). In addition, confirmatory evidence for presence of absence of these genes in the 12 *Pseudomonas* type strains (Table S1) was sought by PCR targeting *phlD* (Figure S1). PCR was carried out in 50-μL volumes containing 3% DMSO, 1 × buffer (Roche Applied Science, Meylan, France), 1.5 mM MgCl_2_, 100 μM of each dNTP, 1 μM of primers B2BF and BPR4 (Almario et al., 2013a), 1.8 U of Taq Expand High Fidelity DNA polymerase (Roche Applied Science) and 1 μL of template genomic DNA. The cycling program included 3 min at 94°C, 30 amplification cycles of 1 min at 94°C, 1 min at 62°C and 1 min at 72°C, and an elongation step of 3 min at 72°C. In the absence of amplification, the *phlD* negative status of the strains was verified by conducting the PCR at lower primer hybridization temperatures (60, 58, 56, and 54°C instead of 62°C). PCR products were purified (MinElute PCR purification kit; Qiagen, Courtaboeuf, France) and both strands were sequenced (LGC Genomics, Berlin, Germany). The sequence pairs were trimmed and assembled with BioEdit v.7.0 (Hall, 1999), and their identity levels with *phlD* were determined using the BlastN algorithm and the nr Nucleotide Sequence Database.

### DAPG quantification by HPLC

DAPG production was measured by HPLC (Bonsall et al., 1997). Samples were analyzed with an Agilent 1200 series HPLC (Agilent Technologies, Santa Carla, USA) equipped with a degasser (G132A), a quaternary pump module (G1311A), an automatic sampler (G1329A), and a diode array detector (DAD G1315B). Separation of compounds was performed at room temperature with a C18 reverse-phase column. For each sample, 20 μL were injected and eluted at 1 mL min^−1^ using a step-by-step gradient increasing acetonitrile proportion in water: the gradient started at 40% of acetonitrile over 4 min and rose from 40 to 64% in 7.5 min, then reached 75% at 16.5 min, and ended at 100% at 18.5 min (100% maintained for 3 min before decreasing to 40% for 5 min). Chromatograms were recorded at 270 nm (maximum of absorbance of DAPG). The Chemstation Agilent software was used for integration of chromatograms, and quantitation of DAPG was done according to a standard curve with a chemical standard (Sigma-Aldrich). This experiment was done three times independently.

### Phylogenetic analysis of *Pseudomonas* strains

**Figure 1:**
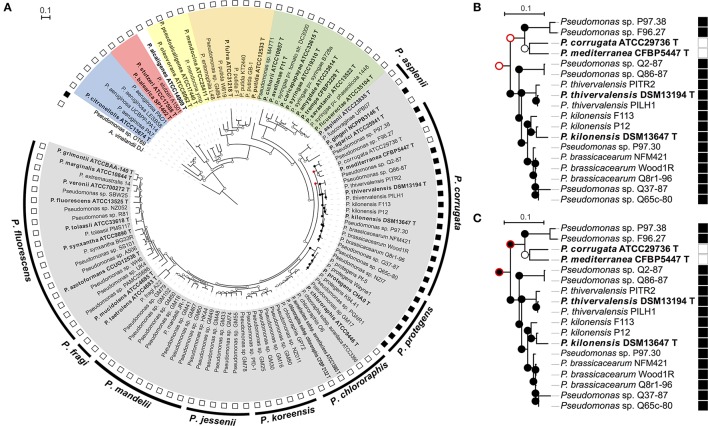
Ancestral state character reconstruction by Maximum Parsimony of the gains and losses of the *phl* operon. **(A)** Phylogenetic *rrs*-*gyrB*-*rpoB* tree of selected *Pseudomonas* strains in which each node indicates presence (black circle) or absence of *phl* operon (*phlACBD*) (white circle). The red circle indicates multiple, equally-parsimonious scenarios that are shown in **(B,C)**. The outer ring represents the subgroups defined in the “*Pseudomonas fluorescens*” group according to Mulet et al. (2010), the second ring correspond to the presence (black square) or the absence (white square) of the *phl* cluster. The different *Pseudomonas* groups are highlighted in color: Gray color, “*P. chlororaphis*” group; Green, “*P. syringae*” group; Purple, “*P. putida*” group; Yellow, “*P. oleovorans*” group; Red, “*P. stutzeri*” group; Blue, “*P. aeruginosa*” group. Fluorescent *Pseudomonas* strains of uncertain taxonomic status are written as “sp.” and those misclassified were renamed based on *rrs-rpoD-gyrB* phylogeny and ANI data (See Tables S2–S7). **(B,C)** The two equally parsimonious scenarios inferred by ancestral reconstruction for the most recent common ancestor of the “*P. corrugata*” subgroup. Contradicting nodes are denoted by a red outer circle. Strains P97.30, Q37-87, and Q65c-80 likely belong to *P. brassicacearum* but are portrayed as *Pseudomonas* sp. in the absence of ANI data.

The reconstruction of the *Pseudomonas* species phylogenetic tree was based on *rpoB, gyrB*, and *rrs* housekeeping genes. Nucleotide sequences were retrieved from Genbank (Table S1), then aligned using MUSCLE (Edgar, 2004) and informative positions were selected using Gblocks with relaxed parameters (Castresana, 2000). Alignments of the three genes were then concatenated and used to compute a Maximum Likelihood tree using PhyML (Guindon et al., 2010) with the following parameters: HKY85 model (Hasegawa et al., 1985), SPR topology search, estimation of invariants sites, and robustness assessment with 1,000 bootstraps. The phylogenetic reconstruction of the *phlACBD* operon was done using the same procedure. Fluorescent *Pseudomonas* strains of uncertain taxonomic status are written as “sp.” and those misclassified (e.g., strain F113) were renamed based on *rrs-rpoD-gyrB* phylogeny and on average nucleotide identity (ANI) data (Richter and Rosselló-Móra, 2009) (Figure 1A, Figure S2 and Tables S2–S7).

### Ancestral state reconstruction in *Pseudomonas*

Mesquite (Maddison and Maddison, 2010) was used to infer the presence or absence of the *phlACBD* operon at each node of the *Pseudomonas* species phylogenetic tree based on its distribution in terminal taxa. Analyses were done using the Maximum Parsimony method, which allows reconstructing ancestral states by minimizing character change events along a phylogenetic tree.

### Genetic environment of the *phl* cluster in *Pseudomonas* genomes

Sequenced genomes of *phl*^+^ strains (Table S1) available in april 2016 were recovered and the 40-kb flanking genetic environment of the *phl* cluster (*phlACBD* operon and genes *phlE, phlI, phlH, phlG*, and *phlF*), encompassing the upstream and downstream regions, was analyzed using online JGI Integrated Microbial Genomes tools (https://img.jgi.doe.gov/).

The 40-kb flanking regions were screened for signs of genome instability based on G + C content analysis in Artemis (Rutherford et al., 2000), tRNA detection using tRNAscan-SE (Schattner et al., 2005), insertion sequence detection using ISfinder (Kichenaradja et al., 2010) and genomic island prediction using Island Viewer (Langille and Brinkman, 2009).

## Results

### Distribution of the *phl* operon among *Pseudomonas* strains

A total of 24 of 111 *Pseudomonas* strains displayed *phlACBD* genes. As expected, *phl*^+^ *Pseudomonas* strains clustered either in the “*P. corrugata*” or the “*P. protegens*” subgroup of the “*P. fluorescens*” group (Figure 1A and Figure S2). Within the “*P. corrugata*” subgroup, two clades could be distinguished. A first clade comprised *phl*^+^ strains from the multilocus phylogenetic groups A, B, C, and D defined in Frapolli et al. (2007), as well as *phl*^+^ type strains *P. brassicacearum*^T^*, P. kilonensis*^T^*, and P. thivervalensis*^T^. A second clade within the “*P. corrugata*” subgroup comprised *phl*^+^ strains (*Pseudomonas* sp. F96.27 and P97.38) from multilocus phylogenetic group E along with *phl*^−^ *P. mediterranea*^T^ *and P. corrugata*^T^. Within the “*P. protegens*” subgroup, all the strains were harboring a *phl* operon and belonged to the multilocus phylogenetic group F defined in Frapolli et al. (2007). When the capacity to produce DAPG was tested in representative strains (including type strains) from the “*P. chlororaphis*” group, i.e., (i) *P. fluorescens*^T^, *P. tolaasii*^T^, *P. marginalis*^T^ (“*P. fluorescens*” subgroup), (ii) *P. chlororaphis* subsp. *aurantiaca*^T^, *P. chlororaphis* subsp. *aureofaciens*^T^, *P. chlororaphis*^T^ (“*P. chlororaphis*” subgroup), (iii) *P. brassicacearum* ^T^, *P. kilonensis*^T^, *P. kilonensis* F113 (formerly *P. “fluorescens”* F113), *P. mediterranea*^T^, *P. thievervalensis*^T^, *P. corrugata* ^T^ (“*P. corrugata*” subgroup), (iv) *P. protegens* ^T^, *P. protegens* Pf-5 (“*P. protegens*” subgroup), and (v) *P. asplenii*^T^, *P. fuscovaginae*^T^, *P. rhodesiae*^T^, *P. gingeri*^T^ (“*P. asplenii*” subgroup), only pseudomonads harboring the *phlACBD* genes could produce DAPG (Figure S3). Besides the “*P. corrugata*” and “*P. protegens*” subgroups, two other *Pseudomonas* strains possess a *phl* operon. The first one, *P. gingeri* NCPPB3146 ^T^, is a member of the “*P. fluorescens*” group but does not belong to any defined subgroup (Mulet et al., 2010), whereas the second one, *Pseudomonas* sp. OT69 is a *Pseudomonas* strain clustering outside of the “*P. fluorescens*” group (Figure 1A).

### Phylogeny of *phl*^+^ *Pseudomonas*

The *phlACBD*-based phylogenetic tree was substantially congruent with the one inferred from concatenated housekeeping genes *rpoB, gyrB*, and *rrs* since the differences between the two trees were restricted to lower-level branches and were not supported by high bootstrap scores (Figure S2). The *phlACBD*-based phylogenetic tree presented four clades. A first *phlACBD* clade included strains belonging to the “*P. corrugata*” subgroup (Figure S2), with the same separation found previously (Frapolli et al., 2007) between the multilocus phylogenetic groups A, B, C, and D on one side and group E on the other. A second *phl* clade comprised strains belonging to the “*P. protegens*” subgroup (multilocus group F). Finally, two clades harboring a single strain each, i.e., *P. gingeri* NCPPB3146 ^T^ and *Pseudomonas* sp. OT69, which were found in more ancestral branches of the *phl* operon tree (Figure S2).

The four clades of *phl*^+^ *Pseudomonas* described above were clearly distinguished in the *rrs-gyrB-rpoB* tree (Figure 1A and Figure S2). Indeed, strains from multilocus groups A to D and type strains *P. brassicacearum*^T^, *P. kilonensis*^T^, and *P. thivervalensis*^T^ formed one clade and were separated from group-E strains of the same “*P. corrugata*” subgroup, while strains from multilocus group F (i.e., “*P. protegens*” subgroup) clustered together, and *P. gingeri*^T^ remained on a single branch forming an outgroup.

### Ancestral state reconstruction for the *phl* operon

The polyphyletic distribution of *phlACBD* suggests that this operon could have undergone multiple losses and/or horizontal transfer events. Ancestral state reconstruction for the *phl* operon supported a scenario with multiple acquisitions. Indeed, this operon was independently acquired by *Pseudomonas* sp. OT69, *P. gingeri* NCPPB3146^T^ and by the most recent common ancestor (MRCA) of the “*P. protegens*” subgroup (Figure 1A), and it was acquired possibly once or twice by the MRCA of the “*P. corrugata*” subgroup. Two parsimonious scenarios are possible to explain acquisition(s) of the *phl* operon by species of the “*P. corrugata*” subgroup. In the first scenario, two independent acquisitions can be proposed, i.e., by the MRCA of *Pseudomonas* strains P97.38 and F96.27 and the MRCA of the clade comprised of *P. thivervalensis, P. kilonensis, P. brassicacearum*, and *Pseudomonas* strains Q2-87 and Q65c-80 (Figure 1B). The second scenario is an ancestral acquisition by the MRCA of the “*P. corrugata*” subgroup and a subsequent loss in the MRCA of *P. corrugata* ATCC29736/*P. mediterranea* CFBP5447 (Figure 1C).

**Figure 2:**
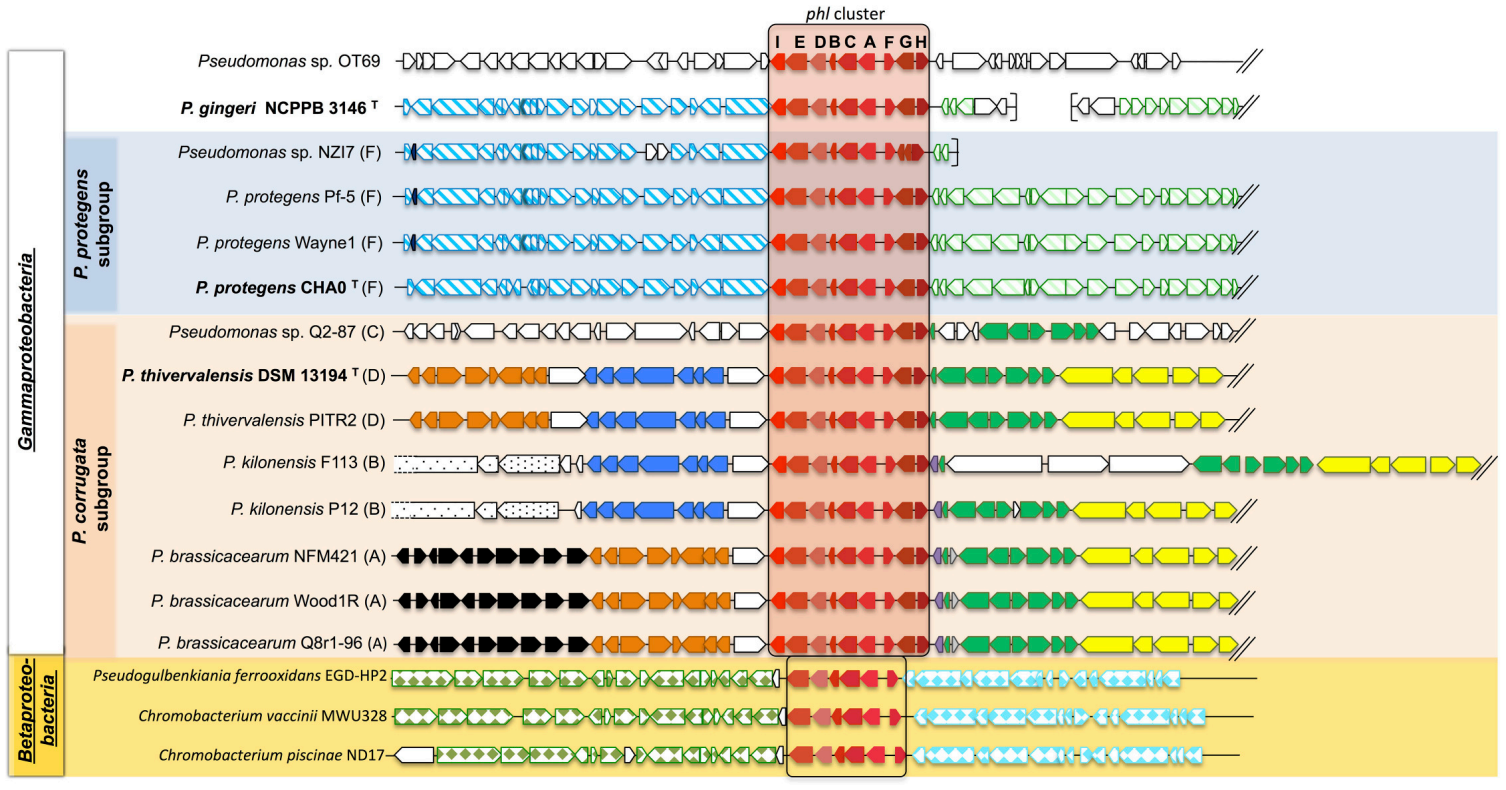
Synteny of the genomic regions flanking the *phl* cluster among *Pseudomonas* genomes. The *phl* cluster is colored in red. Letters correspond to the name of each *phl* gene. Type strains are shown in bold, and letters following the strain name correspond to the multilocus phylogenetic groups defined in Frapolli et al. (2007). Each group of genes of homologous synteny downstream (at the left of the *phl* cluster) or upstream (at the right of the *phl* cluster) is presented using the same color/pattern, and strain-specific genes using white color. Brackets indicate the beginning or the end of a contig. The unclassified strains are positioned in a subgroup according to the phylogeny obtained in Figure 1. Fluorescent *Pseudomonas* strains of uncertain taxonomic status are written as “sp.” and those misclassified were renamed based on *rrs-rpoD-gyrB* phylogeny and ANI data (See Tables S2–S7).

Seeking the nr database for *phl* homologs, the gene cluster containing *phlFACBDE* was also retrieved outside the *Pseudomonas* clade. This was the case for several Betaproteobacteria, i.e., *Chromobacterium vaccinii* MWU328, *C. vaccinii* MWU205, *Chromobacterium piscinae* ND17, and *Pseudogulbenkiania ferrooxidans* EGD-HP2 (Figure 2 and Figure S4).

### Genomic regions flanking the *phl* cluster in *Pseudomonas*

The genomic environment of the *phl* cluster (*phlACBD* operon and regulation/transport genes *phlE, phlF, phlG, phlH*, and *phlI)* was investigated 20 kb upstream and 20 kb downstream of the *phl* cluster (Figure 2). Apart from the *phl* cluster itself, no gene was common to all strains in the 40 kb surrounding region (Figure 2). However, the synteny showed higher conservation for strains belonging to the same *Pseudomonas* subgroup.

In the “*P. corrugata*” subgroup, comparing the available genomes, the 40 kb genomic region surrounding the *phl* cluster showed high plasticity since only seven genes were conserved amongst all the strains analyzed (on the right of the *phl* cluster in Figure 2). Five different groups of genes presented synteny between “*P. corrugata*” subgroup strains. However, the distribution and position of these gene clusters were identical only within each multilocus group. Indeed, bacteria from the multilocus group A encompassing *P. brassicacearum* NFM421, Wood1R, and Q8r1-96 shared the exact same gene order in the vicinity of the *phl* cluster (Figure 2). Only multilocus group B (*P. kilonensis* P12 and F113) presented internal dissimilarity upstream of the *phl* cluster. *P. kilonensis* F113 showed an insertion of three genes for synthesis of an insecticidal toxin complex protein (cable pili-associated adhesin protein, virulence plasmid A protein, and an insecticidal toxin).

In the “*P. protegens*” subgroup (corresponding to multilocus group F), the region surrounding the *phl* cluster showed complete synteny (Figure 2). This was also the case for the downstream region of *P. gingeri* even though this strain falls outside the “*P. protegens*” subgroup.

Only *Pseudomonas* sp. OT69 harbored a specific *phl* genomic environment sharing no similarity with any other *phl*^+^ strain (Figure 2).

Insertion sequences, genes coding for tRNA, integrases or transposases, or other genes associated with genome instability were not found in the vicinity of the *phl* cluster in the genomes of the “*P. fluorescens*” group. In addition, there was no marked difference in G + C content when comparing the *phl* cluster to the rest of the genome (data not shown). Outside the “*P. fluorescens*” group, *Pseudomonas* sp. OT69 displayed a 29,375-bp putative genomic island of 28 genes (including the *phl* cluster) with a codon usage different from the rest of the genome, based on Hidden Markov Model analysis, suggesting recent horizontal transfer of this gene cluster.

## Discussion

So far, analysis of the evolutionary history of the *phl* cluster has focused mainly on the phylogenetic relations among established *phl*^+^ pseudomonads, omitting closely-related *Pseudomonas* species of uncertain *phl* status. Here, we investigated the distribution of the *phl* cluster among a broader taxonomic range of *Pseudomonas* species, then established phylogenetic relations between *phl*^+^ and *phl*^−^ *Pseudomonas*, and explored the genomic context of the *phl* cluster.

Within the “*P. corrugata*” subgroup, besides *P. protegens* and *P. brassicacearum* (for which DAPG production was documented; Ortet et al., 2011; Ramette et al., 2011; Bruto et al., 2014), the *phl* operon was also found in *P. kilonensis* and *P. thivervalensis* types strains, a possibility already suggested by the observation that closely-related pseudomonads displayed *phlD* (Frapolli et al., 2012). The *phl* operon is absent from the *P. fluorescens* species, which conflicts with the many literature reports indicating otherwise, but in which *P. fluorescens* was used as a convenient generic term for ill-defined fluorescent pseudomonads with biocontrol properties rather than an established taxonomic status *per se* (Couillerot et al., 2009). In the same way, an incorrect species assignment was detected for the strain *P*. “*alcaligenes*” OT69, which appears far from the *P. alcaligenes* type strain in the phylogenetic tree (Figure 1). Overall, the *phl*^+^ *Pseudomonas* did not form a monophyletic group, as the *phl* operon was found (i) in some but not all species of the “*P. corrugata*” subgroup, (ii) in all strains of the “*P. protegens*” subgroup, and (iii) in *P. gingeri* and *Pseudomonas* sp. OT69 but not in pseudomonads closely related to any of the latter.

Moynihan et al. (2009) proposed that the *phl* cluster was present in the last common ancestor of the “*P. fluorescens”* group but was only retained in one branch, giving rise to a divide between a *phl*^+^ and a *phl*^−^ group of fluorescent *Pseudomonas* and thus a paraphyletic distribution. However, our results based on a higher number of species (type strains) and related strains do not support this hypothesis. On the contrary, ancestral character reconstructions favored multiple independent acquisitions rather than one ancestral event followed by vertical inheritance. First, ancestral state reconstruction (Figure 1) indicates that the *phl* cluster was absent from the last common ancestor of the “*P. fluorescens”* group, and was probably acquired three times in this group, i.e., by the MRCA of the “*P. corrugata*” subgroup, by the MRCA of the “*P. protegens*” subgroup, and by the species *P. gingeri*. Second, *phl*^+^ *Pseudomonas* form a polyphyletic group, in that the *phl* cluster is not present in all descendants of the last common ancestor of *phl*^+^ *Pseudomonas*, suggesting that the *phl* cluster may have undergone several loss and/or horizontal transfer events. Thus, the only uncertainty in the reconstruction was found in the “*P. corrugata”* subgroup where two equally-likely scenarios may be proposed. However, the scenario suggesting an acquisition by the MRCA of the “*P. corrugata*” subgroup and a subsequent loss by the MRCA of *P. corrugata* ATCC29736^T^/*P. mediterranea* CFBP5447^T^ can be favored on the basis of two arguments. First, large scale genomic analysis suggests that gene loss is more common and likely than genomic acquisitions (Wolf and Koonin, 2013). Second, *P. corrugata* ATCC29736^T^ and *P. mediterranea* CFBP5447^T^ are the only species in the “*P. corrugata”* subgroup known to be plant pathogens, causing pith necrosis in tomato, eggplant, pepper, and tobacco (Catara et al., 2002). Since DAPG is able to elicit plant defenses via ISR pathways (Iavicoli et al., 2003), the *phl* cluster may have been counter-selected and lost in these species during their adaptation to a phytopathogenic lifestyle. In the case of *P. syringae*, gene loss is a major mechanism of adaptation to pathogenicity (Mithani et al., 2010), and indeed it was recently shown that *P. corrugata* and *P. mediterranea* genomes specialized into pathogenicity via gene loss events (Trantas et al., 2015).

While signs of recent horizontal transfers were only found in the distant strain *Pseudomonas* sp. OT69, no signature was identified in the other *Pseudomonas* strains, supporting the ancestral acquisition by each clade. Recent literature proposed that the *phlACBD* operon was presumably formed in *Pseudomonas* through the acquisition of *phlACB* from Archaea and *phlD* from another unclear origin, possibly *Streptomyces, Vibrio* (Kidarsa et al., 2011), or even plants (Cook et al., 1995; Ramette et al., 2001). The current study showed that the *phlACBD* operon (and genes *phlE* and *phlF*) is also present in the Betaproteobacteria *C. vaccinii* and *Pseudogulbenkiania ferrooxidans* EGD-HP2, suggesting a more complex evolutionary history (Figures 2 and Figure S4).

Thus, we have shown that the *phl*^+^ *Pseudomonas* form a polyphyletic group. This is an important finding to take into consideration when assessing the ecological significance of DAPG^+^ populations, which have been proposed as soil health bioindicators because of their contribution to the suppression of plant diseases (Sanguin et al., 2008; Almario et al., 2013a). First, it means that “DAPG-producing bacteria” corresponds to a broader phylogenetic range than usually thought (Sanguin et al., 2008). Second, it raises the possibility of more diversified regulation patterns for DAPG production than in *Pseudomonas* strains investigated so far (Duffy and Défago, 1999; Schnider-Keel et al., 2000). Third, a larger range of DAPG-producing bacteria implies more varied combinations of ecological traits in addition to DAPG production, which are likely to influence the response of these bacteria to environmental factors and their contribution to ecological processes such as plant-disease suppression (Sanguin et al., 2008; Frapolli et al., 2010; Schreiner et al., 2010).

## Author contributions

JA, MB, YM, and DM conceived and designed the experiments. JA performed the experiment. MB performed the ancestral state reconstruction, JV and CP the DAPG HPLC analyses. JV, MB, JA, and DM analyzed the data and JA, JV, YM, and DM wrote the manuscript; all authors contributed to the discussion and approved the final manuscript.

### Conflict of interest statement

The authors declare that the research was conducted in the absence of any commercial or financial relationships that could be construed as a potential conflict of interest.

## Supplementary material

Figure S1 Presence/absence of *phlD* gene in the 12 *Pseudomonas* type strains sought by PCR targeting *phlD*. MW, molecular weight; 1 and 6, negative control; 2, *P. brassicacearum* ^T^; 3, *P. kilonensis* ^T^; 4, *P. thivervalensis* ^T^; 5, *P. mediterranea* ^T^; 7, *P. corrugata* ^T^; 8, *P. protegens* ^T^; 9, 10, and 15, *P. kilonensis* F113 (positive control); 11, *P. chlororaphis* subsp. *aurantiaca* ^T^; 12, *P. chlororaphis* subsp. *aureofaciens* ^T^; 13, *P. chlororaphis* subsp. *chlororaphis* ^T^; 14, *P. tolaasii* ^T^; 16, *P. marginalis* ^T^; 17, *P. chlororaphis* ^T^.

Figure S2 Comparison of species tree of *phl*^+^ *Pseudomonas* strains and *phlACBD* tree for these bacteria. (A) Phylogenetic analysis based on concatenated housekeeping genes *rpoB, gyrB*, and *rrs*. When available, membership to the multilocus phylogenetic groups defined in Frapolli et al. (2007) is given in parenthesis. (B) Phylogenetic analysis of *phl*^+^ *Pseudomonas* based on nucleotide sequences for the operon *phlACBD*. In both panels, the Maximum Likelihood tree was inferred using PhyML model, and nodal robustness was assessed using 1,000 bootstrap replicates. Type strains are shown in bold and written in blue. Fluorescent *Pseudomonas* strains of uncertain taxonomic status are written as “sp.” and those misclassified were renamed based on *rrs-rpoD-gyrB* phylogeny and ANI data (See Tables S2–S7).

Figure S3 DAPG production in *Pseudomonas* type strains. (A) HPLC chromatogram, UV, and mass spectra of DAPG standard. (B) HPLC detection of DAPG in *Pseudomonas* strain supernatants.

Figure S4 Phylogenetic analysis of the *phl* operon for the main *Pseudomonas* subgroups and betaproteobacterial species. When available, membership to the multilocus phylogenetic groups defined in Frapolli et al. (2007) is given in parenthesis. The Maximum Likelihood tree was inferred using PhyML model, and nodal robustness was assessed using 500 bootstrap replicates. The “*P. corrugata*” subgroup is highlighted in orange and the “*P. protegens*” subgroup in blue. Type strains are indicated in bold and red. Fluorescent *Pseudomonas* strains of uncertain taxonomic status are written as “sp.” and those misclassified were renamed based on *rrs-rpoD-gyrB* phylogeny and ANI data (See Tables S2–S7).

Table S1 *Pseudomonas* strains and accession numbers for nucleotidic sequences used in this study.

Table S2 Average nucleotide identity values (calculated using Blast algorithm) for the assignment of uncertain pseudomonads to the *P. protegens* species.

Table S3 Average nucleotide identity values (calculated using MUMmer algorithm) for the assignment of uncertain pseudomonads to the *P. protegens* species.

Table S4 Average nucleotide identity values (calculated using Blast algorithm) for the assignment of uncertain pseudomonads to the *P. chlororaphis* species.

Table S5 Average nucleotide identity values (calculated using MUMmer algorithm) for the assignment of uncertain pseudomonads to the *P. chlororaphis* species.

Table S6 Average nucleotide identity values (calculated using Blast algorithm) for the assignment of uncertain pseudomonads to the *P. brassicacearum, P. kilonensis*, and *P. thivervalensis* species.

Table S7 Average nucleotide identity values (calculated using MUMmer algorithm) for the assignment of uncertain pseudomonads to the *P. brassicacearum, P. kilonensis*, and *P. thivervalensis* species.
