# Supplementary figures and images for: Distribution of 2,4-Diacetylphloroglucinol Biosynthetic Genes among the Pseudomonas spp. Reveals Unexpected Polyphyletism
Source: Front Microbiol. 2017 Jun 30;8:1218. doi: 10.3389/fmicb.2017.01218 (PMC5491608; doi:10.3389/fmicb.2017.01218)

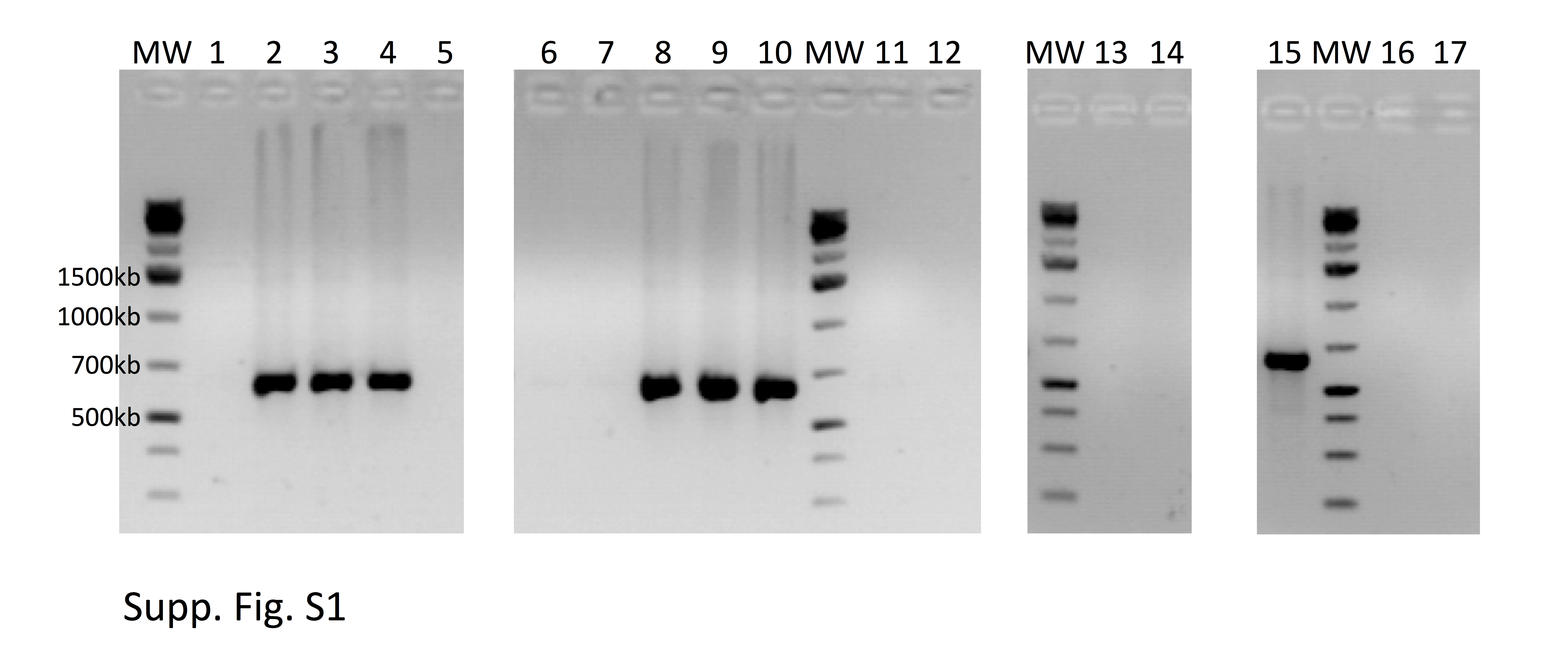

Supplement: Figure S1 — Presence/absence of phlD gene in the 12 Pseudomonas type strains sought by PCR targeting phlD. MW, molecular weight; 1 and 6, negative control; 2, P. brassicacearum T; 3, P. kilonensis T; 4, P. thivervalensis T; 5, P. mediterranea T; 7, P. corrugata T; 8, P. protegens T; 9, 10, and 15, P. kilonensis F113 (positive control); 11, P. chlororaphis subsp. aurantiaca T; 12, P. chlororaphis subsp. aureofaciens T; 13, P. chlororaphis subsp. chlororaphis T; 14, P. tolaasii T; 16, P. marginalis T; 17, P. chlororaphis T. [file Image1.JPEG]

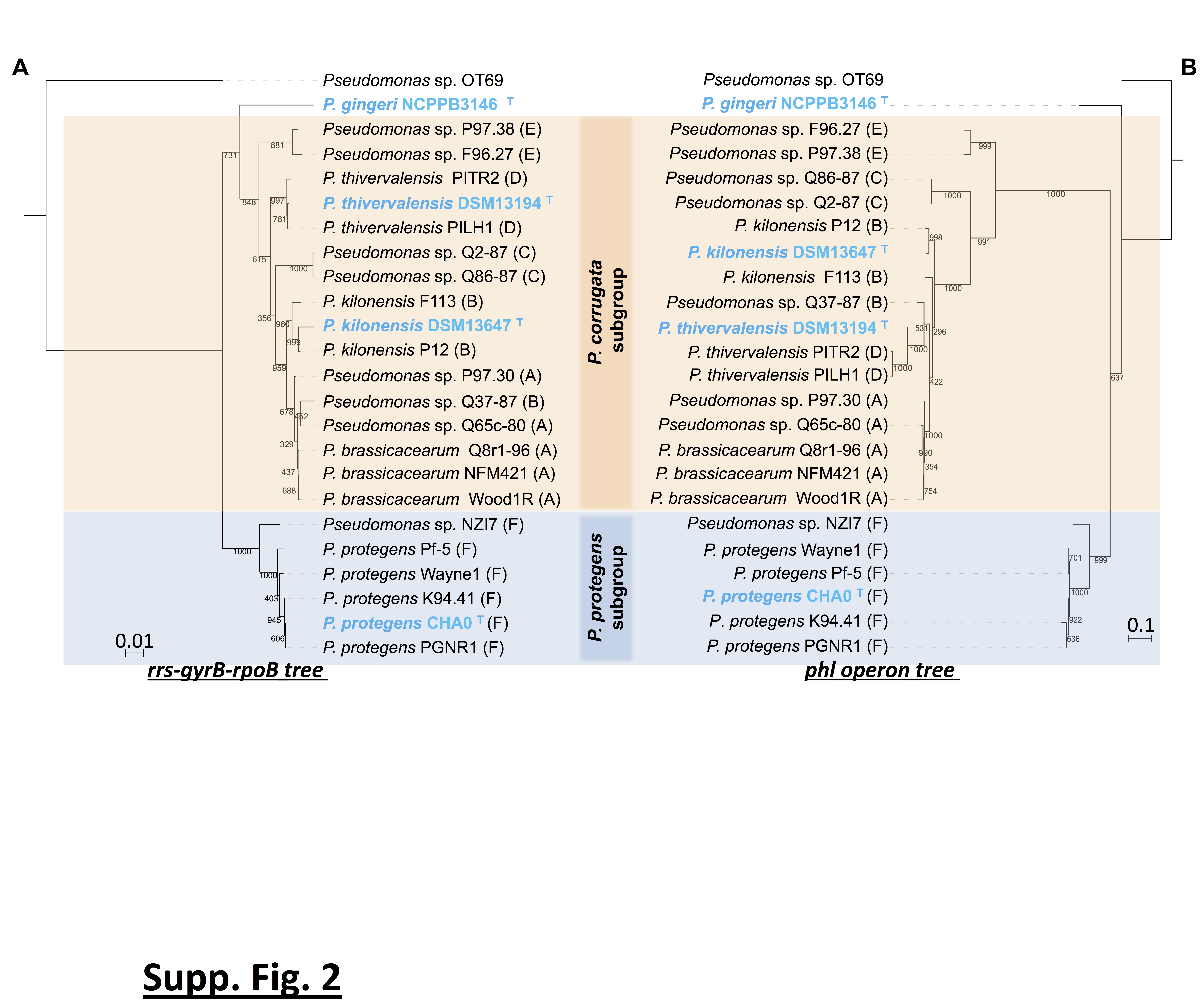

Supplement: Figure S2 — Comparison of species tree of phl+ Pseudomonas strains and phlACBD tree for these bacteria. (A) Phylogenetic analysis based on concatenated housekeeping genes rpoB, gyrB, and rrs. When available, membership to the multilocus phylogenetic groups defined in Frapolli et al. (2007) is given in parenthesis. (B) Phylogenetic analysis of phl+ Pseudomonas based on nucleotide sequences for the operon phlACBD. In both panels, the Maximum Likelihood tree was inferred using PhyML model, and nodal robustness was assessed using 1,000 bootstrap replicates. Type strains are shown in bold and written in blue. Fluorescent Pseudomonas strains of uncertain taxonomic status are written as “sp.” and those misclassified were renamed based on rrs-rpoD-gyrB phylogeny and ANI data (See Tables S2–S7). [file Image2.jpg]

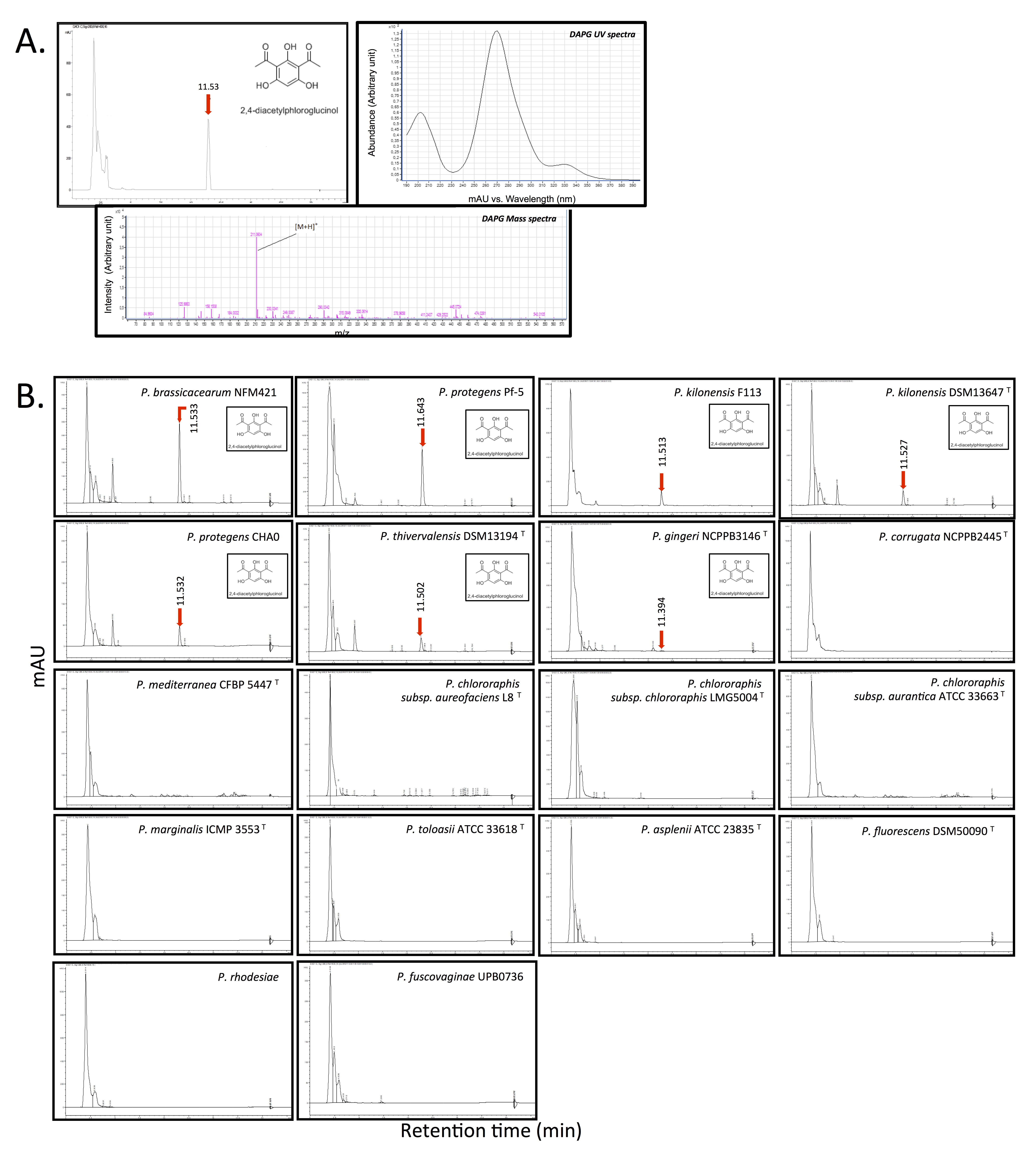

Supplement: Figure S3 — DAPG production in Pseudomonas type strains. (A) HPLC chromatogram, UV, and mass spectra of DAPG standard. (B) HPLC detection of DAPG in Pseudomonas strain supernatants. [file Image3.JPEG]

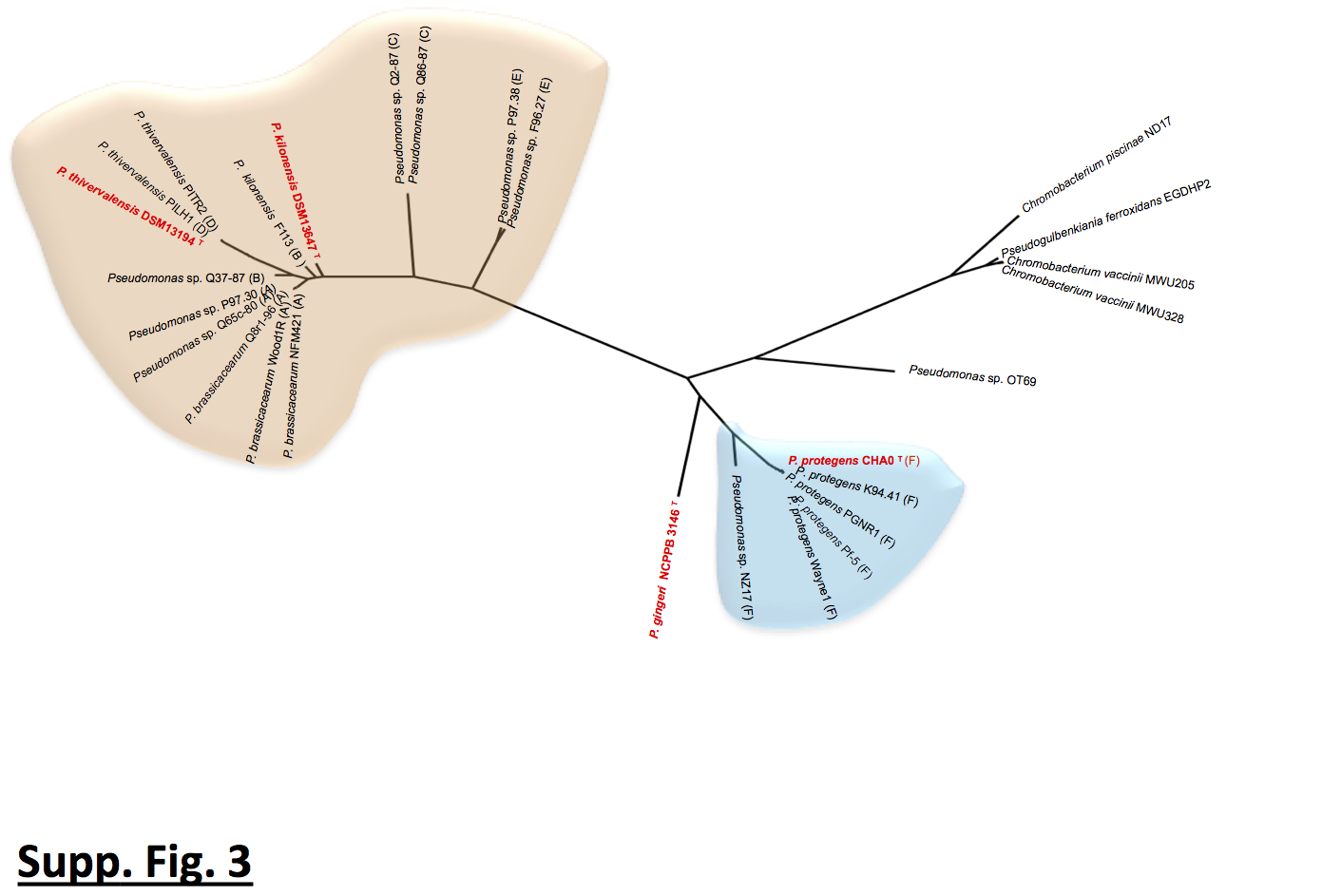

Supplement: Figure S4 — Phylogenetic analysis of the phl operon for the main Pseudomonas subgroups and betaproteobacterial species. When available, membership to the multilocus phylogenetic groups defined in Frapolli et al. (2007) is given in parenthesis. The Maximum Likelihood tree was inferred using PhyML model, and nodal robustness was assessed using 500 bootstrap replicates. The “P. corrugata” subgroup is highlighted in orange and the “P. protegens” subgroup in blue. Type strains are indicated in bold and red. Fluorescent Pseudomonas strains of uncertain taxonomic status are written as “sp.” and those misclassified were renamed based on rrs-rpoD-gyrB phylogeny and ANI data (See Tables S2–S7). [file Image4.JPEG]
