# Supplementary material for: Distribution of 2,4-Diacetylphloroglucinol Biosynthetic Genes among the Pseudomonas spp. Reveals Unexpected Polyphyletism
Source: Front Microbiol. 2017 Jun 30;8:1218. doi: 10.3389/fmicb.2017.01218 (PMC5491608; doi:10.3389/fmicb.2017.01218)
Supplement: Table S1 — Pseudomonas strains and accession numbers for nucleotidic sequences used in this study. [file Table1.DOCX]

**Table S1.** *Pseudomonas* strains and accession numbers for nucleotidic sequences used in this study

|  | **Accession numbers ^a^** | | | | | | | | | | | | |
| --- | --- | --- | --- | --- | --- | --- | --- | --- | --- | --- | --- | --- | --- |
| ***Pseudomonas* strains** | ***phlA*** | ***phlC*** | | ***phlB*** | | ***phlD*** | | ***rpoB*** | | ***gyrB*** | | ***rrs*** | |
| *P. aeruginosa* LESB58 |  |  | |  | |  | | NC_011770.1 | | | | | |
| *P. aeruginosa* PA7 |  |  | |  | |  | | NC_009656.1 | | | | | |
| *P. aeruginosa* PAO1 |  |  | |  | |  | | NC_002516.2 | | | | | |
| *P. aeruginosa* UCBPP-PA14 |  |  | |  | |  | | NC_008463.1 | | | | | |
| *P. agarici* NCPPB2289 |  |  | |  | |  | | NZ_AKBQ00000000.1 | | | | | |
| *P.* *alcaligenes* NBRC 14159 T |  |  | |  | |  | | NZ_BATI00000000.1 | | | | | |
| *P.* *amygdali* CFBP 3205 T |  |  | |  | |  | | NZ_JYHB00000000.1 | | | | | |
| *P.* *asplenii* ATCC 23835 ^T^ |  |  | |  | |  | | AJ717432 | | AB039455 | | NR_040802.1 | |
| *P.* *avellanae* BPIC 631 ^T^ |  |  | |  | |  | | NZ_ATDK00000000.1 | | | | | |
| *P.* *azotoformans* CCUG 12536 ^T^ |  |  | |  | |  | | AJ717458 | | AB039411 | | AB680322 | |
| *P.* *balearica* DSM 6083 ^T^ |  |  | |  | |  | | NZ_CP007511.1 | | | | | |
| *P.* *brassicacearum* NFM421 | NC_015379.1 | | | | | | | | | | | | |
| *P. brassicacearum* Q8r1-96 | NZ_CM001512.1 | | | | | | | | | | | | |
| *P.* *brassicacearum* Wood1R | NZ_CAFF00000000.1 | | | | | | | | | | | | |
| *P.* *caricapapayae* ICMP 2855 ^T^ |  |  | |  | |  | | NZ_LJPW00000000.1 | | | | | |
| *P.* *chl*. subsp*.* *aurantica* ATCC 33663 ^T^ | |  | |  | |  | | AJ717421 | | FN554171 | | NR_112076 | |
| *P.* *chl*. subsp. *aureofaciens* L8 ^T^ |  |  | |  | |  | | NZ_BBQB00000000.1 | | | | | |
| *P.* *chl*. subsp. *chlororaphis* LMG5004 ^T^ | |  | |  | |  | | LHVC00000000.1 | | | | | |
| *P.* *chlororaphis* GP72 |  |  | |  | |  | | NZ_AHAY00000000.1 | | | | | |
| *P.* *chlororaphis* O6 |  |  | |  | |  | | NZ_CM001490.1 | | | | | |
| *P.* *cichorii* NCPPB 943 ^T^ |  |  | |  | |  | | AJ717418 | | AB039434 | | Z76658 | |
| *P.* *citronellolis*  ^T^ |  |  | |  | |  | | FN568270 | | AB039452 | | NR_112069 | |
| *P.* *corrugata* NCPPB2445 ^T^ |  |  | |  | |  | | NZ_LIGR00000000.1 | | | | | |
| *P.* *entomophila* L48 |  |  | |  | |  | | NC_008027.1 | | | | | |
| *P.* *extremaustralis* 14-3 substr. 14-3b | |  | |  | |  | | NZ_AHIP00000000.1 | | | | | |
| *P.* *ficuserectae* ICMP 7848 ^T^ |  |  | |  | |  | | LJQJ00000000.1 | | | | | |
| *P.* *fluorescens* DSM 50090 ^T^ |  |  | |  | |  | | LHVP00000000.1 | | | | | |
| *P.* *fragi* A22 |  |  | |  | |  | | NZ_AHZY00000000.1 | | | | | |
| *P.* *fulva* 12-X |  |  | |  | |  | | NC_015556 | | | | | |
| *P.* *fuscovaginae* UPB0736 |  |  | |  | |  | | NZ_AIEU00000000.1 | | | | | |
| *P.* *gingeri* NCPPB 3146 | NZ_AKBP00000000.1 | | | | | | | | | | | | |
| *P.* *grimontii* ATCC BAA-140 ^T^ |  |  | |  | |  | | AJ717439 | | FN554188 | | AF268029 | |
| *P.* *kilonensis* DSM 13647 ^T^ | LHVH00000000.1 | | | | | | | | | | | | |
| *P.* *kilonensis* F113 | NC_016830.1 | | | | | | | | | | | | |
| *P.* *kilonensis* P12 | LHVG00000000.1 | | | | | | | | | | | | |
| *P.* *mandelii* JR-1 |  |  | |  | |  | | NZ_CP005960.1 | | | | | |
| *P.* *marginalis* ICMP 3553 ^T^ |  |  | |  | |  | | NZ_LKEG00000000.1 | | | | | |
| *P.* *mediterranea* CFBP 5447 ^T^ |  |  | |  | |  | | AUPB00000000.1 | | | | | |
| *P.* *mendocina* NBRC14162 ^T^ |  |  | |  | |  | | NZ_BBQC00000000.1 | | | | | |
| *P.* *mendocina* ymp |  |  | |  | |  | | NC_009439.1 | | | | | |
| *P.* *mucidolens* ATCC4685 ^T^ |  |  | |  | |  | | AJ717427 | | AB039409 | | D84017 | |
| *P.* *oleovorans* ATCC 8062 ^T^ |  |  | |  | |  | | AJ717461 | | AB039396 | | NR_114478 | |
| *P.* *protegens* CHA0 ^T^ | NC_021237.1 | | | | | | | | | | | | |
| *P.* *protegens* K94.41 | LHUU00000000.1 | | | | | | | | | | | | |
| *P.* *protegens* Pf-5 | NC_004129.6 | | | | | | | | | | | | |
| *P.* *protegens* PGNR1 | LHUV00000000.1 | | | | | | | | | | | | |
| *P. protegens* Wayne1 | NZ_CADX00000000.1 | | | | | | | | | | | | |
| *P.* *pseudoalcaligenes* CECT 5344 |  |  | |  | |  | | NZ_HG916826 | | | | | |
| *P.* *putida* F1 |  |  | |  | |  | | NC_009512.1 | | | | | |
| *P.* *putida* GB-1 |  |  | |  | |  | | NC_010322.1 | | | | | |
| *P.* *putida* KT2440 |  |  | |  | |  | | NC_002947.4 | | | | | |
| *P.* *putida* ^T^ |  |  | |  | |  | | NC_021505.1 | | | | | |
| *P.* *putida* W619 |  |  | |  | |  | | NC_010501.1 | | | | | |
| *P.* *savastanoi* ATCC 13522 ^T^ |  |  | |  | |  | | NZ_LJRJ00000000.1 | | | | | |
| *P.* *savastanoi* pv. *phaseolicola* 1448 |  |  | |  | |  | | NC_005773.3 | | | | | |
| *P.* *stutzeri* A1501 |  |  | |  | |  | | NC_009434.1 | | | | | |
| *P.* *stutzeri* CGMCC 1.1803 ^T^ |  |  | |  | |  | | NC_015740.1 | | | | | |
| *P.* *synxantha* ATCC 9890 ^T^ |  |  | |  | |  | | NZ_JYLJ00000000.1 | | | | | |
| *P.* *synxantha* BG33R |  |  | |  | |  | | NZ_CM001514.1 | | | | | |
| *P.* *syringae* ICMP3023 ^T^ |  |  | |  | |  | | NZ_LJRK00000000.1 | | | | | |
| *P.* *syringae* pv. *syringae* B728a |  |  | |  | |  | | NC_007005.1 | | | | | |
| *P.* *syringae* pv. *tomato* DC3000 |  |  | |  | |  | | NC_004578.1 | | | | | |
| *P.* *taetrolens* ATCC 4683 ^T^ |  |  | |  | |  | | NZ_JYLA00000000.1 | | | | | |
| *P.* *thivervalensis* DSM 13194 ^T^ | LHVE00000000.1 | | | | | | | | | | | | |
| *P.* *thivervalensis* PILH1 | EF424694 | | EF424734 | | EF424728 | | AJ278810 | | DQ458649 | | DQ458584 | | DQ453824 |
| *P.* *thivervalensis* PITR2 | LHVF00000000.1 | | | | | | | | | | | | |
| *P.* *tolaasii* ATCC 33618 ^T^ |  |  | |  | |  | | AJ717467 | | FN645137 | | AJ308317 | |
| *P.* *tolaasii* PMS117 |  |  | |  | |  | | NZ_AJXG00000000.1 | | | | | |
| *P.* *tremae* ICMP9151 ^T^ |  |  | |  | |  | | NZ_LJRO00000000.1 | | | | | |
| *P.* *veronii* ATCC 700272 ^T^ |  |  | |  | |  | | NZ_JYLL00000000.1 | | | | | |
| *Pseudomonas* sp. A506 |  |  | |  | |  | | NC_017911.1 | | | | | |
| *Pseudomonas* sp. F96.27 | EF424697 | EF424737 | | EF424722 | | EF424744 | | DQ458641 | | DQ458576 | | DQ453817 | |
| *Pseudomonas* sp. GM102 |  |  | |  | |  | | NZ_AKJB00000000.1 | | | | | |
| *Pseudomonas* sp. GM16 |  |  | |  | |  | | NZ_AKJV00000000.1 | | | | | |
| *Pseudomonas* sp. GM18 |  |  | |  | |  | | NZ_AKJT00000000.1 | | | | | |
| *Pseudomonas* sp. GM25 |  |  | |  | |  | | NZ_AKJQ00000000.1 | | | | | |
| *Pseudomonas* sp. GM30 |  |  | |  | |  | | NZ_AKJP00000000.2 | | | | | |
| *Pseudomonas* sp. GM33 |  |  | |  | |  | | NZ_AKJO00000000.1 | | | | | |
| *Pseudomonas* sp. GM41 |  |  | |  | |  | | NZ_AKJN00000000.2 | | | | | |
| *Pseudomonas* sp. GM48 |  |  | |  | |  | | NZ_AKJM00000000.1 | | | | | |
| *Pseudomonas* sp. GM49 |  |  | |  | |  | | NZ_AKJL00000000.1 | | | | | |
| *Pseudomonas* sp. GM50 |  |  | |  | |  | | NZ_AKJK00000000.1 | | | | | |
| *Pseudomonas* sp. GM55 |  |  | |  | |  | | NZ_AKJJ00000000.1 | | | | | |
| *Pseudomonas* sp. GM60 |  |  | |  | |  | | NZ_AKJI00000000.1 | | | | | |
| *Pseudomonas* sp. GM67 |  |  | |  | |  | | NZ_AKJH00000000.1 | | | | | |
| *Pseudomonas* sp. GM74 |  |  | |  | |  | | NZ_AKJG00000000.1 | | | | | |
| *Pseudomonas* sp. GM78 |  |  | |  | |  | | NZ_AKJF00000000.1 | | | | | |
| *Pseudomonas* sp. GM79 |  |  | |  | |  | | NZ_AKJE00000000.1 | | | | | |
| *Pseudomonas* sp. GM80 |  |  | |  | |  | | NZ_AKJD00000000.1 | | | | | |
| *Pseudomonas* sp. GM84 |  |  | |  | |  | | NZ_AKJC00000000.1 | | | | | |
| *Pseudomonas* sp. HK44 |  |  | |  | |  | | NZ_AFOY00000000.2 | | | | | |
| *Pseudomonas* sp. M47T1 |  |  | |  | |  | | NZ_AJWX00000000.1 | | | | | |
| *Pseudomonas* sp. NZ011 |  |  | |  | |  | | NZ_AJXJ00000000.1 | | | | | |
| *Pseudomonas* sp. NZ052 |  |  | |  | |  | | NZ_AJXH00000000.1 | | | | | |
| *Pseudomonas* sp. NZI7 | AJXF00000000.1 | | | | | | | | | | | | |
| *Pseudomonas* sp. OT69 | NZ_ATCP00000000.1 | | | | | | | | | | | | |
| *Pseudomonas* sp. P97.30 | EF424704 | EF424740 | | EF424725 | | EF424747 | | DQ458646 | | DQ458581 | | DQ453822 | |
| *Pseudomonas* sp. P97.38 | LHVJ00000000.1 | | | | | | | | | | | | |
| *Pseudomonas* sp. PAMC 25886 |  |  | |  | |  | | NZ_AHHC00000000.1 | | | | | |
| *Pseudomonas* sp. Pf0-1 |  |  | |  | |  | | NC_007492.2 | | | | | |
| *Pseudomonas* sp. Q2-87 | NZ_CM001558.1 | | | | | | | | | | | | |
| *Pseudomonas* sp. Q37-87 | EF424703 | EF424731 | | EF424729 | | AY928641 | | DQ458651 | | DQ458587 | | AJ417069 | |
| *Pseudomonas* sp. Q65c-80 | EF424696 | EF424730 | | EF424718 | | AJ278807 | | DQ458652 | | DQ458588 | | AJ417074 | |
| *Pseudomonas* sp. Q86-87 | HQ395489 | HQ395517 | | HQ395503 | | EF554356 | | EF044527 | | EF044475 | | EF044361 | |
| *Pseudomonas* sp. R81 |  |  | |  | |  | | NZ_AHZN00000000.1 | | | | | |
| *Pseudomonas* sp. SBW25 |  |  | |  | |  | | NC_012660.1 | | | | | |
| *Pseudomonas* sp. SS101 |  |  | |  | |  | | NZ_CM001513.1 | | | | | |
| *Pseudomonas* sp. UK4 |  |  | |  | |  | | NZ_CP008896.1 | | | | | |
| *Pseudomonas* sp. WH6 |  |  | |  | |  | | NZ_CM001025.1 | | | | | |

^a^ **in grey accession to genomes in white target genes when genomes are unavailable**
