# Supplementary material for: Distribution of 2,4-Diacetylphloroglucinol Biosynthetic Genes among the Pseudomonas spp. Reveals Unexpected Polyphyletism
Source: Front Microbiol. 2017 Jun 30;8:1218. doi: 10.3389/fmicb.2017.01218 (PMC5491608; doi:10.3389/fmicb.2017.01218)
Supplement: Table S2 — Average nucleotide identity values (calculated using Blast algorithm) for the assignment of uncertain pseudomonads to the P. protegens species. [file Table2.DOCX]

|  | ***P. protegens* CHA0 ^T^** | *P. protegens* Pf-5 | *P. protegens* Wayne1 | *Pseudomonas* sp. NZI7 | *P.* *protegens* K94.41 | *P.* *protegens* PGNR1 |
| --- | --- | --- | --- | --- | --- | --- |
| ***P. protegens* CHA0 ^T^** ^a^ | - | **98.65 ^b^** | **98.66** | 89.24 | **98.57** | **100.00** |
|  |  | *[95.10]* **^c^** | *[92.31]* | *[78.49]* | *[95.73]* | *[99.80]* |
| *P. protegens* Pf-5 | **98.55** | - | **98.97** | 89.20 | **98.33** | **98.55** |
|  | *[92.42]* |  | *[90.65]* | *[76.10]* | *[91.88]* | *[92.40]* |
| *P. protegens* Wayne1 | **98.70** | **99.08** | - | 89.26 | **98.50** | **98.70** |
|  | *[92.39]* | *[93.74]* |  | *[76.79]* | *[91.73]* | *[92.40]* |
| *Pseudomonas* sp. NZI7 | 89.09 | 89.03 | 89.10 | - | 89.19 | 89.09 |
|  | *[82.03]* | *[82.04]* | *[80.28]* |  | *[82.14]* | *[82.02]* |
| *P. protegens* K94.41 | **98.53** | **98.37** | **98.42** | 89.30 | - | **98.53** |
|  | *[94.23]* | *[93.08]* | *[90.08]* | *[77.37]* |  | *[94.24]* |
| *P. protegens* PGNR1 | **100.00** | **98.65** | **98.68** | 89.23 | **98.58** | - |
|  | *[99.89]* | *[95.15]* | *[92.45]* | *[78.44]* | *[95.69]* |  |
| **^a^** The type strain is indicated in bold.  **^b^** ANI values indicated in green are above the threshold for the prokaryotic species definition (96% according to Richter and Rosselló-Móra, 2009).  **^c^** The values indicated in brackets correspond to the percentage of length aligned during the ANI calculation. Only values beyond 70% of aligned sequenced should be considered. | | | | | | |

**Table S2.** Average nucleotide identity values (calculated using Blast algorithm) for the assignment of uncertain pseudomonads to the *P.* *protegens* species.
