# Supplementary material for: Distribution of 2,4-Diacetylphloroglucinol Biosynthetic Genes among the Pseudomonas spp. Reveals Unexpected Polyphyletism
Source: Front Microbiol. 2017 Jun 30;8:1218. doi: 10.3389/fmicb.2017.01218 (PMC5491608; doi:10.3389/fmicb.2017.01218)
Supplement: Table S3 — Average nucleotide identity values (calculated using MUMmer algorithm) for the assignment of uncertain pseudomonads to the P. protegens species. [file Table3.docx]

**Table S3.** Average nucleotide identity values (calculated using MUMmer algorithm) for the assignment of uncertain pseudomonads to the *P.* *protegens* species.

|  | ***P. protegens* CHA0 ^T^** | *P. protegens* Pf-5 | *P. protegens* Wayne1 | *Pseudomonas* sp. NZI7 | *P. protegens* K94.41 | *P. protegens* PGNR1 |
| --- | --- | --- | --- | --- | --- | --- |
| ***P. protegens* CHA0 ^T a^** | - | **98.87 ^b^** | **98.68** | 90.91 | **99.00** | **99.99** |
|  |  | *[95.59]* **^c^** | *[95.08]* | *[79.04]* | *[95.54]* | *[99.98]* |
| *P. protegens* Pf-5 | **98.87** | - | **99.05** | 90.88 | **98.81** | **98.86** |
|  | *[92.88]* |  | *[93.27]* | *[76.61]* | *[91.98]* | *[92.88]* |
| *P. 'fluorescens'* Wayne1 | **98.68** | **99.05** | - | 90.76 | **98.66** | **98.68** |
|  | *[94.84]* | *[95.78]* |  | *[78.50]* | *[93.76]* | *[94.84]* |
| *Pseudomonas* sp. NZI7 | 90.91 | 90.87 | 90.76 | - | 90.89 | 90.91 |
|  | *[79.35]* | *[79.19]* | *[79.00]* |  | *[79.73]* | *[79.35]* |
| *P. protegens* K94.41 | **99.00** | **98.82** | **98.66** | 90.89 | - | **99.00** |
|  | *[94.15]* | *[93.30]* | *[92.62]* | *[78.33]* |  | *[94.15]* |
| *P. protegens* PGNR1 | **99.99** | **98.86** | **98.68** | 90.91 | **99.00** | - |
|  | *[100.00]* | *[95.60]* | *[95.09]* | *[78.98]* | *[95.52]* |  |

**^a^** The type strain is indicated in bold.

**^b^** ANI values indicated in green are above the threshold for the prokaryotic species definition (96% according to Richter and Rosselló-Móra, 2009)

**^c^** The values indicated in brackets correspond to the percentage of length aligned during the ANI calculation. Only values beyond 70% of aligned sequenced should be considered.
