# Supplementary material for: Distribution of 2,4-Diacetylphloroglucinol Biosynthetic Genes among the Pseudomonas spp. Reveals Unexpected Polyphyletism
Source: Front Microbiol. 2017 Jun 30;8:1218. doi: 10.3389/fmicb.2017.01218 (PMC5491608; doi:10.3389/fmicb.2017.01218)
Supplement: Table S4 — Average nucleotide identity values (calculated using Blast algorithm) for the assignment of uncertain pseudomonads to the P. chlororaphis species. [file Table4.DOCX]

**Table S4.** Average nucleotide identity values (calculated using Blast algorithm) for the assignment of uncertain pseudomonads to the *P.* *fluorescens* species.

|  | ***P. fluorescens* DSM 50090 ^T^** | *Pseudomonas* sp. NZ011 | *Pseudomonas* sp. A506 | *Pseudomonas* sp. SS101 | *Pseudomonas* sp. Pf0-1 | *Pseudomonas* sp. SBW25 | *Pseudomonas* sp. NZ052 | *Pseudomonas* sp. HK44 | *Pseudomonas* sp. WH6 |
| --- | --- | --- | --- | --- | --- | --- | --- | --- | --- |
| ***P. fluorescens***  **DSM 50090 ^T a^** | - | 79.34 **^b^** | 85.60 | 85.81 | 79.81 | 86.61 | 86.42 | 80.06 | 85.83 |
|  |  | *[59.55]* **^c^** | *[71.61]* | *[71.12]* | *[60.07]* | *[73.71]* | *[73.91]* | *[51.57]* | *[69.01]* |
| *Pseudomonas* sp. NZ011 | 79.31 | - | 79.14 | 79.20 | 85.99 | 79.68 | 79.84 | 81.61 | 79.56 |
|  | *[57.17]* |  | *[55.91]* | *[55.98]* | *[74.43]* | *[56.66]* | *[56.78]* | *[53.24]* | *[54.56]* |
| *Pseudomonas* sp. A506 | 85.87 | 79.55 | - | 94.70 | 80.13 | 86.20 | 86.19 | 80.15 | 86.02 |
|  | *[76.23]* | *[61.94]* |  | *[87.22]* | *[62.11]* | *[74.67]* | *[74.95]* | *[53.43]* | *[72.13]* |
| *Pseudomonas* sp. SS101 | 85.87 | 79.41 | 94.45 | - | 79.98 | 86.33 | 86.15 | 80.17 | 86.09 |
|  | *[73.89]* | *[60.64]* | *[84.88]* |  | *[60.46]* | *[72.52]* | *[73.20]* | *[51.19]* | *[69.18]* |
| *Pseudomonas* sp.  Pf0-1 | 79.77 | 86.06 | 79.79 | 79.81 | - | 80.19 | 80.30 | 82.17 | 80.10 |
|  | *[60.62]* | *[77.42]* | *[58.77]* | *[58.69]* |  | *[60.43]* | *[60.48]* | *[55.90]* | *[58.40]* |
| *Pseudomonas* sp. SBW25 | 86.17 | 79.39 | 85.45 | 85.72 | 79.90 | - | 88.56 | 80.13 | 86.73 |
|  | *[71.53]* | *[56.98]* | *[67.97]* | *[68.05]* | *[58.40]* |  | *[74.26]* | *[48.93]* | *[70.21]* |
| *Pseudomonas* sp.  NZ052 | 85.90 | 79.87 | 85.36 | 85.44 | 80.04 | 88.42 | - | 79.98 | 86.16 |
|  | *[70.89]* | *[57.08]* | *[67.86]* | *[68.52]* | *[58.69]* | *[73.71]* |  | *[48.85]* | *[66.08]* |
| *Pseudomonas* sp. HK44 | 79.96 | 81.65 | 79.85 | 79.91 | 82.11 | 80.25 | 80.14 | - | 80.22 |
|  | *[55.06]* | *[59.21]* | *[53.32]* | *[52.69]* | *[59.57]* | *[54.29]* | *[54.14]* |  | *[54.88]* |
| *Pseudomonas* sp. WH6 | 85.78 | 79.70 | 85.70 | 85.82 | 80.04 | 87.12 | 86.65 | 80.16 | - |
|  | *[69.98]* | *[58.02]* | *[68.72]* | *[68.13]* | *[59.42]* | *[73.84]* | *[69.69]* | *[52.69]* |  |

**^a^** The type strain is indicated in bold.

**^b^** ANI values indicated in light grey should not be considered because below the minimum alignment length (70%).

**^c^** The values indicated in brackets correspond to the percentage of length aligned during the ANI calculation. Only values beyond 70% of aligned sequenced should be considered.
