# Supplementary material for: Distribution of 2,4-Diacetylphloroglucinol Biosynthetic Genes among the Pseudomonas spp. Reveals Unexpected Polyphyletism
Source: Front Microbiol. 2017 Jun 30;8:1218. doi: 10.3389/fmicb.2017.01218 (PMC5491608; doi:10.3389/fmicb.2017.01218)
Supplement: Table S5 — Average nucleotide identity values (calculated using MUMmer algorithm) for the assignment of uncertain pseudomonads to the P. chlororaphis species. [file Table5.docx]

**Table S5.** Average nucleotide identity values (calculated using MUMmer algorithm) for the assignment of uncertain pseudomonads to the *P.* *fluorescens* species.

|  | ***P. fluorescens* DSM 50090 ^T^** | *Pseudomonas* sp. NZ011 | *Pseudomonas* sp. A506 | *Pseudomonas* sp. SS101 | *Pseudomonas* sp. Pf0-1 | *Pseudomonas* sp. SBW25 | *Pseudomonas* sp. NZ052 | *Pseudomonas* sp. HK44 | *Pseudomonas* sp. WH6 |
| --- | --- | --- | --- | --- | --- | --- | --- | --- | --- |
| ***P. fluorescens***  **DSM 50090 ^T a^** | - | 85.44 **^b^** | 87.97 | 88.09 | 85.54 | 88.45 | 88.28 | 85.67 | 88.02 |
|  |  | *[35.31]* **^c^** | *[66.81]* | *[67.07]* | *[38.00]* | *[71.21]* | *[71.68]* | *[33.15]* | *[65.91]* |
| *Pseudomonas* sp. NZ011 | 85.44 | - | 85.40 | 85.43 | 88.23 | 85.51 | 85.75 | 86.09 | 85.60 |
|  | *[33.03]* |  | *[32.05]* | *[32.17]* | *[70.29]* | *[34.48]* | *[34.75]* | *[39.60]* | *[32.88]* |
| *Pseudomonas* sp. A506 | 87.97 | 85.40 | - | **95.34** | 85.71 | 88.28 | 88.18 | 85.65 | 88.17 |
|  | *[71.80]* | *[36.98]* |  | *[88.32]* | *[40.19]* | *[71.43]* | *[72.25]* | *[34.23]* | *[69.25]* |
| *Pseudomonas* sp. SS101 | 88.09 | 85.43 | **95.34** | - | 85.66 | 88.46 | 88.26 | 85.68 | 88.28 |
|  | *[69.69]* | *[35.85]* | *[85.78]* |  | *[38.84]* | *[70.13]* | *[70.74]* | *[33.17]* | *[67.18]* |
| *Pseudomonas* sp.  Pf0-1 | 85.54 | 88.22 | 85.71 | 85.66 | - | 85.83 | 85.62 | 86.36 | 85.67 |
|  | *[38.20]* | *[74.81]* | *[37.34]* | *[37.38]* |  | *[39.78]* | *[41.13]* | *[44.18]* | *[39.25]* |
| *Pseudomonas* sp. SBW25 | 88.45 | 85.51 | 88.28 | 88.46 | 85.83 | - | 90.40 | 85.79 | 88.78 |
|  | *[67.72]* | *[35.21]* | *[63.40]* | *[64.17]* | *[37.93]* |  | *[74.17]* | *[32.19]* | *[68.43]* |
| *Pseudomonas* sp.  NZ052 | 88.29 | 85.74 | 88.18 | 88.26 | 85.62 | 90.39 | - | 85.76 | 88.68 |
|  | *[66.78]* | *[34.84]* | *[62.78]* | *[63.34]* | *[38.47]* | *[72.58]* |  | *[31.58]* | *[62.75]* |
| *Pseudomonas* sp. HK44 | 85.67 | 86.09 | 85.64 | 85.67 | 86.38 | 85.78 | 85.76 | - | 85.77 |
|  | *[35.28]* | *[44.83]* | *[33.71]* | *[33.88]* | *[46.87]* | *[35.98]* | *[35.96]* |  | *[35.72]* |
| *Pseudomonas* sp. WH6 | 88.01 | 85.61 | 88.16 | 88.28 | 85.67 | 88.78 | 88.69 | 85.78 | - |
|  | *[66.78]* | *[35.71]* | *[65.30]* | *[65.49]* | *[39.71]* | *[72.66]* | *[68.12]* | *[34.04]* |  |

**^a^** The type strain is indicated in bold.

**^b^** ANI values indicated in light grey should not be considered because below the minimum alignment length (70%). ANI values indicated in maroon are between 95% and 96%, which provides plausible taxonomic information, but they are (just) below the 96% reference threshold for the prokaryotic species definition (according to Richter and Rosselló-Móra, 2009).

**^c^** The values indicated in brackets correspond to the percentage of length aligned during the ANI calculation. Only values beyond 70% of aligned sequenced should be considered.
