# Supplementary material for: Distribution of 2,4-Diacetylphloroglucinol Biosynthetic Genes among the Pseudomonas spp. Reveals Unexpected Polyphyletism
Source: Front Microbiol. 2017 Jun 30;8:1218. doi: 10.3389/fmicb.2017.01218 (PMC5491608; doi:10.3389/fmicb.2017.01218)
Supplement: Table S6 — Average nucleotide identity values (calculated using Blast algorithm) for the assignment of uncertain pseudomonads to the P. brassicacearum, P. kilonensis, and P. thivervalensis species. [file Table6.DOCX]

**Table S6.** Average nucleotide identity values (calculated using Blast algorithm) for the assignment of uncertain pseudomonads to the *P.* *brassicacearum*, *P.* *kilonensis* and *P.* *thivervalensis* species.

|  | *P.*  *brassicacearum* NFM421 | *P. brassicacearum* Wood1R | *P.*  *brassicacearum* Q8r1-96 | ***P.***  ***kilonensis* DSM13647 ^T^** | *P.*  *kilonensis* F113 | *P.*  *kilonensis* P12 | ***P.***  ***thivervalensis* DSM13194 ^T^** | *P.*  *thivervalensis* PITR2 |
| --- | --- | --- | --- | --- | --- | --- | --- | --- |
| *P. brassicacearum* NFM421 | - | **99.09 ^a^** | **99.24** | 93.66 | 94.17 | 93.68 | 90.89 | 90.81 |
|  |  | *[84.25]* **^b^** | *[92.87]* | *[77.66]* | *[82.55]* | *[77.75]* | *[78.12]* | *[77.97]* |
| *P. brassicacearum* Wood1R | **99.10** | - | **99.03** | 93.98 | 94.37 | 93.89 | 91.22 | 91.19 |
|  | *[93.15]* |  | *[92.10]* | *[79.88]* | *[84.44]* | *[79.92]* | *[81.76]* | *[81.74]* |
| *P. brassicacearum* Q8r1-96 | **99.49** | **99.27** | - | 93.86 | 94.36 | 93.89 | 91.06 | 91.14 |
|  | *[95.51]* | *[85.51]* |  | *[80.03]* | *[84.44]* | *[80.04]* | *[80.48]* | *[80.15]* |
| ***P. kilonensis* DSM13647 ^T c^** | 94.15 | 94.59 | 94.16 | - | **95.03** | **98.52** | 91.57 | 91.49 |
|  | *[82.09]* | *[75.49]* | *[81.89]* |  | *[83.69]* | *[92.15]* | *[80.77]* | *[79.99]* |
| *P. kilonensis* F113 | 94.28 | 94.46 | 94.26 | 94.67 | - | 94.70 | 91.18 | 91.19 |
|  | *[82.09]* | *[75.58]* | *[81.54]* | *[78.69]* |  | *[77.97]* | *[77.19]* | *[77.30]* |
| *P. kilonensis* P12 | 94.26 | 94.55 | 94.22 | **98.53** | **95.06** | - | 91.60 | 91.56 |
|  | *[82.10]* | *[75.47]* | *[82.06]* | *[92.09]* | *[83.02]* |  | *[79.25]* | *[79.12]* |
| ***P. thivervalensis* DSM13194 ^T^** | 91.11 | 91.41 | 91.09 | 91.34 | 91.19 | 91.29 | - | **98.24** |
|  | *[80.49]* | *[75.93]* | *[80.55]* | *[79.12]* | *[80.33]* | *[77.71]* |  | *[92.16]* |
| *P. thivervalensis* PITR2 | 91.06 | 91.35 | 91.12 | 91.20 | 91.18 | 91.17 | **98.24** | - |
|  | *[78.29]* | *[73.96]* | *[78.27]* | *[76.06]* | *[78.29]* | *[75.47]* | *[89.70]* |  |

**^a^** ANI values indicated in green are above the threshold for the prokaryotic species definition (96% according to Richter and Rosselló-Móra, 2009), those in maroon are between 95% and 96%.

**^b^** The values indicated in brackets correspond to the percentage of length aligned during the ANI calculation. Only value beyond 70% of aligned sequenced should be considered.

**^c^** Type strains are indicated in bold.
