# Supplementary material for: Distribution of 2,4-Diacetylphloroglucinol Biosynthetic Genes among the Pseudomonas spp. Reveals Unexpected Polyphyletism
Source: Front Microbiol. 2017 Jun 30;8:1218. doi: 10.3389/fmicb.2017.01218 (PMC5491608; doi:10.3389/fmicb.2017.01218)
Supplement: Table S7 — Average nucleotide identity values (calculated using MUMmer algorithm) for the assignment of uncertain pseudomonads to the P. brassicacearum, P. kilonensis, and P. thivervalensis species. [file Table7.docx]

**Table S7.** Average nucleotide identity values (calculated using MUMmer algorithm) for the assignment of uncertain pseudomonads to the *P.* *brassicacearum*, *P.* *kilonensis* and *P.* *thivervalensis* species.

|  | *P.*  *brassicacearum* NFM421 | *P. brassicacearum* Wood1R | *P.*  *brassicacearum* Q8r1-96 | ***P.***  ***kilonensis* DSM13647 ^T^** | | *P.*  *kilonensis* F113 | | *P.*  *kilonensis* P12 | | ***P.***  ***thivervalensis* DSM13194 ^T^** | *P.*  *thivervalensis*  PITR2 | |
| --- | --- | --- | --- | --- | --- | --- | --- | --- | --- | --- | --- | --- |
| *P. brassicacearum* NFM421 | - | **99.60 ^a^** | **99.58** | **95.08** | | **95.19** | | **95.06** | | 92.42 | 92.39 | |
|  |  | *[90.74]* **^b^** | *[92.97]* | *[79.21]* | | *[84.23]* | | *[79.23]* | | *[79.62]* | *[79.21]* | |
| *P. brassicacearum* Wood1R | **99.60** | - | **99.59** | **95.38** | | **95.38** | | **95.35** | | 92.56 | 92.52 | |
|  | *[92.48]* |  | *[91.39]* | *[79.15]* | | *[83.99]* | | *[78.99]* | | *[80.40]* | *[80.23]* | |
| *P. brassicacearum* Q8r1-96 | **99.58** | **99.59** | - | **95.07** | | **95.19** | | **95.05** | | 92.40 | 92.40 | |
|  | *[96.31]* | *[92.82]* |  | *[81.90]* | *[86.78]* | | *[81.96]* | | *[82.42]* | | | *[82.03]* |
| ***P. kilonensis* DSM13647 ^T c^** | **95.08** | **95.38** | **95.07** | - | **96.06** | | **98.74** | | 92.85 | | | 92.78 |
|  | *[85.46]* | *[82.94]* | *[85.21]* |  | *[86.20]* | | *[93.71]* | | *[83.76]* | | | *[82.68]* |
| *P. kilonensis* F113 | **95.20** | **95.38** | **95.19** | **96.06** | - | | **96.04** | | 92.68 | | | 92.60 |
|  | *[84.31]* | *[82.33]* | *[83.81]* | *[80.00]* |  |  | *[79.45]* | | *[78.95]* | | | *[78.95]* |
| *P. kilonensis* P12 | **95.06** | **95.35** | **95.06** | **98.74** | **96.04** | | - | | 92.81 | | | 92.76 |
|  | *[85.50]* | *[82.79]* | *[85.29]* | *[93.71]* | *[85.59]* | |  |  | *[82.05]* | | | *[81.58]* |
| ***P. thivervalensis* DSM13194 ^T^** | 92.42 | 92.56 | 92.41 | 92.85 | 92.68 | | 92.82 | | - | | | **98.72** |
|  | *[82.86]* | *[81.78]* | *[82.76]* | *[81.04]* | *[81.88]* | | *[79.47]* | |  |  |  | *[93.05]* |
| *P. thivervalensis* PITR2 | 92.39 | 92.52 | 92.40 | 92.78 | 92.61 | | 92.77 | | **98.72** | | | - |
|  | *[80.42]* | *[79.56]* | *[80.35]* | *[77.89]* | *[79.69]* | | *[77.06]* | | *[90.56]* | | |  |

**^a^** ANI values indicated in green are above the threshold for the prokaryotic species definition (96% according to Richter and Rosselló-Móra, 2009), those in maroon are between 95% and 96%

**^b^** The values indicated in brackets correspond to the percentage of length aligned during the ANI calculation. Only value beyond 70% of aligned sequenced should be considered.

**^c^** Type strains are indicated in bold.
